# A Proximity Ligation Method to Detect Proteins Bound to Single-Stranded DNA after DNA End Resection at DNA Double-Strand Breaks

**DOI:** 10.3390/mps5010003

**Published:** 2021-12-29

**Authors:** Faith C. Fowler, Jessica K. Tyler

**Affiliations:** 1 Department of Pharmacology, Weill Cornell Graduate School of Medical Sciences, New York, NY 10065, USA; faf2008@med.cornell.edu; 2 Department of Pathology and Laboratory Medicine, Weill Cornell Medicine, New York, NY 10065, USA

**Keywords:** single-stranded DNA, proximity ligation assay, DNA end resection, double-strand break repair

## Abstract

After a DNA double-strand break, cells utilize either non-homologous end joining or homologous recombination to repair the broken DNA ends. Homologous recombination requires extensive nucleolytic processing of one of the DNA strands, resulting in long stretches of 3′ single-strand DNA overhangs. Typically, single-stranded DNA is measured using immunofluorescence microscopy to image the foci of replication protein A, a single-stranded DNA-binding protein. Microscopy analysis of bromodeoxyuridine foci under nondenaturing conditions has also been used to measure single-stranded DNA. Here, we describe a proximity ligation assay which uses genome-wide bromodeoxyuridine incorporation to label single-stranded DNA in order to measure the association of a protein of interest with single-stranded DNA. This method is advantageous over traditional foci analysis because it is more direct and specific than traditional foci co-localization microscopy methods, uses only one color channel, and can reveal protein-single-stranded DNA interactions that are rare and potentially undetectable using traditional microscopy methods. We show here the association of replication protein A and bromodeoxyuridine as proof-of-concept.

## 1. Introduction

The DNA damage response is a network of cellular pathways that sense, signal, and repair DNA lesions. Surveillance proteins that monitor DNA integrity can activate cell cycle checkpoints and DNA repair pathways in response to DNA damage to prevent the generation of potentially deleterious mutations. DNA double-strand breaks (DSBs) are particularly deleterious lesions which, if left unrepaired or are repaired incorrectly, can lead to chromosomal translocations and deletions, cell death, and diseases such as cancer [1]. Cells undergo two distinct mechanisms of DSB repair—non-homologous end joining (NHEJ), where the broken DNA ends are ligated together, or homologous recombination (HR), which uses the sister chromatid as a template for repair.

During HR, the broken DNA ends are rapidly bound by the MRE11-RAD50-NBS1 (MRN) complex (MRE11-RAD50-XRS2 in *S. cerevisiae*) [2,3,4]. At DSBs, MRE11 and CtIP initiate short-range DNA end resection, which is then extended by EXO1 and BLM-DNA2, leading to long stretches of 3′ single-stranded DNA (ssDNA) [5,6,7,8]. This ssDNA is subsequently coated with replication protein A (RPA), which stabilizes and protects the ssDNA from degradation [9]. Subsequently, RPA is replaced with RAD51 recombinase, forming a nucleofilament that can scan the genome for a homologous template to achieve accurate repair [10,11].

Studying DNA end resection, RPA/RAD51 loading, and homology search has proven challenging in mammalian systems, in part because repair by NHEJ is the preferred repair pathway and because the amount of ssDNA generated during DNA end resection is minimal compared to double-stranded DNA (dsDNA) in the genome [12,13]. Because of this, much of what is known about DNA end resection was discovered in budding yeast or demonstrated in vitro.

In mammalian cells, methods to analyze DNA end resection either directly measure ssDNA around inducible DSBs using qPCR or genome-wide sequencing, measure RPA as a proxy for ssDNA using flow cytometry or immunofluorescence microscopy, or measure BrdU under nondenaturing conditions using flow cytometry or immunofluorescence microscopy [13,14,15,16,17]. These techniques have been useful in studies of factors that influence DNA end resection, but are not ideal for studying interactions between proteins and ssDNA.

Our research interest in protein-ssDNA interactions led us to develop this method which combines nondenaturing BrdU immunofluorescence microscopy after DSBs with a proximity ligation assay (PLA). If a DSB undergoes DNA end resection, under nondenaturing conditions, BrdU is exposed and available for anti-BrdU antibody binding; however, BrdU incorporated into dsDNA is not. Using this assay, the interaction between a protein of interest and ssDNA can be assessed using fluorescent microscopy while using one color channel. Additionally, this protocol explains how DAPI staining of the nucleus can be used to parse the cell cycle, which is useful when trying to differentiate replication during the S phase from DSB repair in the G_2_ phase. We present here the association of RPA and ssDNA as proof-of-concept of this assay.

## 2. Experimental Design

Here, we describe the procedure using U-2 OS cells grown for two doubling times in media containing bromodeoxyuridine (BrdU), followed by permeabilization and fixation suitable for U-2 OS cells. This protocol can be adapted to different cell lines after optimization of the permeabilization and fixation steps. The proximity ligation procedure itself can be finished in one day. Useful controls include cells without BrdU, cells with no DNA damage, and cells with no primary antibody included.

The use of a fluorescent microscope with built-in software capable of counting foci drastically reduces analysis time and increases the number of cells that can be included in the analysis, as well as reduces bias introduced when counting foci by eye. If access to a microscope such as the BioTek Lionheart LX is not feasible, images taken on any microscope can be processed and opened in ImageJ and foci can be counted using thresholding.

**Figure 1:**
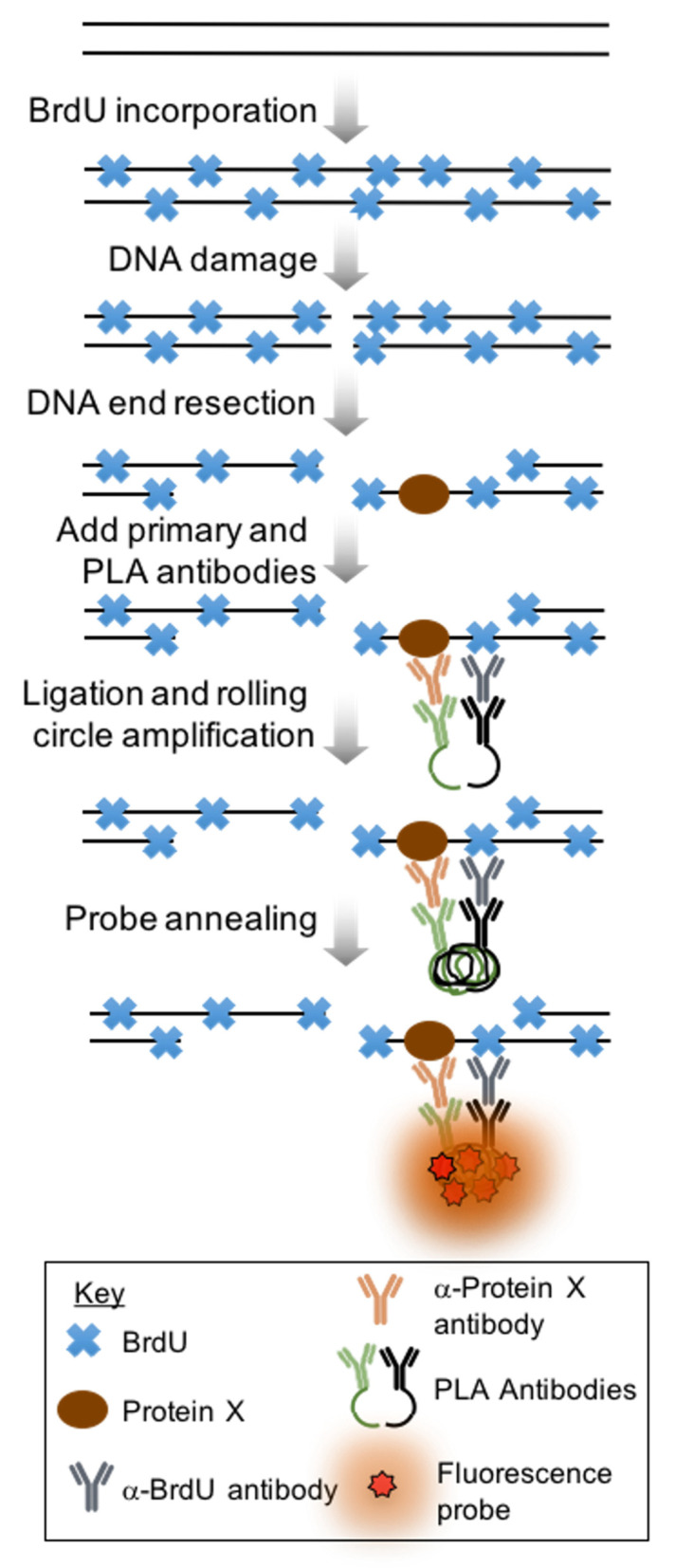
Schematic of proximity ligation assay. First cells are grown for two doubling times in media containing BrdU. Then, a DNA damaging agent such as bleomycin or irradiation is introduced to the cells to generate DSBs, some of which will undergo DNA end resection. Primary antibodies against BrdU and a protein of interest are added to the samples, then cells are incubated with host-specific antibodies conjugated with oligos. These complementary oligos are, next, ligated together. After ligation, a fluorescent probe is annealed and the signal is amplified.

The general outline of the experiment is as follows (Figure 1):

### 2.1. Materials

- 6 cm tissue culture dish (TPP, MIDSCI, Valley Park, MO, USA, Cat. No.: TP93060);
- 24 well tissue culture plates (TPP, MIDSCI, Valley Park, MO, USA, Cat. No.:TP92424;
- 12 mm round glass cover slips (Electron Microscopy Sciences, Hatfield, PA, USA, Cat. No.: 72196-12);
- 0.01% Poly-L-Lysine (Sigma-Aldrich, St. Louis, MO, USA, Cat. No.: A-005-C);
- U-2 OS cells (ATCC, Manassas, VA, USA, Cat. No.: HTB-96);
- BrdU (BD Biosciences, Franklin Lakes, NJ, USA, Cat. No.: 550891);
- Bleomycin sulfate (Enzo Life Sciences, Farmingdale, NY, USA, Cat. No.: BML-AP302-0010);
- Triton-X;
- Formaldehyde;
- 10X PBS (Lonza, Thermo Fisher Scientific, Waltham, MA, USA, Cat. No.: BMA51226);
- Purified mouse anti-BrdU clone 3D4 (BD Biosciences, Franklin Lakes, NJ, USA, Cat. No.: 555627);
- Rabbit anti-RPA70 (Cell Signaling Technology, Danvers, MA, USA, Cat. No.: 2267).
- Duolink^®^ In Situ PLA^®^ Probe Anti-Rabbit PLUS (Sigma-Aldrich, St. Louis, MO, USA, Cat. No.: DUO92002);
- Duolink^®^ In Situ PLA^®^ Probe Anti-Mouse MINUS (Sigma-Aldrich, St. Louis, MO, USA, Cat. No.: DUO92004);
- Duolink^®^ In Situ Detection Reagents Red (Sigma-Aldrich, St. Louis, MO, USA, Cat. No.: DUO92008);
- Duolink^®^ In Situ Wash Buffers, Fluorescence (Sigma-Aldrich, St. Louis, MO, USA, Cat. No.: DUO82049);
- Aluminum foil;
- Nuclease-free water;
- Frosted Microscope Slides (Fisherbrand, Thermo Fisher Scientific, Waltham, MA USA, Cat. No.: 12-550-343);
- Prolong Gold Antifade Mountant with DAPI (Life Technologies, Thermo Fisher Scientific, Waltham, MA, USA, Cat. No.: P36931).

### 2.2. Equipment

- 37 °C, 5% CO_2_ incubator;
- Tissue culture hood;
- Rocker;
- Forceps;
- BioTek Lionheart LX Automated Microscope with Gen5 Software (BioTek Instruments, Winooski, VT, USA);
- Microsoft Excel Software;
- GraphPad Prism Software (GraphPad Software, San Diego, CA, USA);
- ImageJ Software (https://imagej.nih.gov/ij/ (accessed on 25 November 2021));
- CellProfiler Software (www.cellprofiler.org (accessed on 25 November 2021));
- X-ray Irradiator (Rad Source Technologies, Buford, GA, USA, Cat. No.: RS 2000).

## 3. Procedure

### 3.1. Cell Culture (3 Days)

1. Optional: perform siRNA knockdowns of a protein of interest if evaluating effect of protein loss on DNA end resection. For example, transfect cells with 5 nM siControl, siRad51, and siCtIP, and incubate for 48–72 h before moving on to step 2.
2. Seed 500,000 U-2 OS cells in a 6 cm dish with media containing 10 μM BrdU and place in incubator;
3. Approximately 48 h later, place the appropriate number of poly-L-lysine coated glass coverslips into the wells of a 24-well plate;
4. Seed 75,000 U-2 OS cells treated with BrdU onto coverslips and incubate for 18–24 h;
5. Induce DNA damage by either treating cells with 40 μg/mL bleomycin or 20 Gray irradiation and allow to recover for 1–8 h.

### 3.2. Cell Pre-Extraction and Fixation (65 min)

1. Wash cells 3× for 5 min in 1X PBS on a rocker at room temperature;
2. Pre-extract cells with cold 0.2% Triton-X in PBS for 5 min on a rocker at room temperature;
3. Wash cells 3× for 5 min in 1× PBS on a rocker at room temperature;
4. Fix cells with 4% formaldehyde in PBS for 15 min on a rocker at room temperature;
5. Wash cells 3× for 5 min in 1× PBS on a rocker at room temperature.

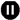
**PAUSE STEP** Fixed cells can be stored at 4 °C in PBS for up to one week.

### 3.3. Primary Antibody Incubation (1 h Plus Overnight)

1. Incubate cells with 5 drops of Duolink Blocking Solution at 37 °C for 1 h;
2. Dilute primary antibodies in included antibody diluent 1:500;
3. Incubate cells in primary antibody overnight at 4 °C on a rocker.

### 3.4. Proximity Ligation Assay (~4 h 15 min Plus Overnight)

1. Wash cells 3× for 5 min in Wash Buffer A on a rocker at room temperature;
2. Dilute oligo-conjugated mouse minus and rabbit plus secondary antibodies 1:5 in included antibody diluent;
3. Incubate cells at 37 °C for 1 h;
4. Wash cells 3× for 5 min in Wash Buffer A on a rocker at room temperature;
5. Dilute thawed ligation buffer 1:5 in nuclease-free water, then dilute ligase 1:40;
6. Incubate cells with ligation mixture at 37 °C for 30 min;
7. Wash cells 3× for 5 min in Wash Buffer A on a rocker at room temperature;
8. Dilute thawed amplification buffer 1:5 in nuclease-free water, then dilute polymerase 1:80;
9. Incubate cells in amplification mixture for 100 min at 37 °C;

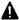
**CRITICAL STEP** Protect from light, wrap plates in foil.

10. Wash cells 2× for 10 min with Wash Buffer B, then 1× for 1 min with 0.01× Wash Buffer B;
11. Add 7 μL of Prolong Gold Antifade with DAPI onto a glass slide;
12. Carefully pick up cover slip from the well using forceps and place cell-side down onto glass slide;
13. Allow cover slips to cure at least overnight in the dark.

### 3.5. Imaging and Analysis

1. Take pictures of slides using DAPI and mCherry channels. At least 100 cells/5 different fields should be imaged. If using the BioTek Lionheart with Gen5 software, pictures of a large area of the slide can be taken and stitched together to create one picture (montage);
2. If desired, quantify DAPI fluorescence using Gen5 software, ImageJ, or CellProfiler [18].

If using Gen5, please refer to the cited technical note for an example of measuring DAPI content [19]:

- Perform image preprocessing to subtract background fluorescence;
- Adjust primary mask (DAPI) fluorescence threshold and cell size to create appropriate primary mask that accurately circles each nucleus;
- Measure total (integral) fluorescence of each nuclei.

Cells can be divided into G_0_/G_1_, S, and G_2_/M phase using the following equation:

G_0_/G_1_ boundary = Average DAPI Fluorescence − (0.5 × (Standard Deviation))
G_2_/M boundary = Average DAPI Fluorescence + (0.5 × (Standard Deviation))
S = Values within −/+ 0.5 × Standard Deviation

Note: this is a rough cell cycle approximation based on S phase accounting for ~1/3 of the cell cycle time and +/− 0.5 × (Standard Deviation) of a normal distribution equaling ~38% of a population. More elegant cell cycle analysis using DAPI quantification can be performed using directions in the cited references [20,21];

3. Count PLA foci manually or with a program such as Gen5, CellProfiler, or ImageJ.

Note: example of spot counting in ImageJ can be found here: https://microscopy.duke.edu/guides/count-nuclear-foci-ImageJ (accessed on 25 November 2021);

4. If desired, mask PLA foci and measure fluorescence intensity with Gen5, CellProfiler, or ImageJ;
5. Enter data to be analyzed into an analysis software such as Graphpad Prism, such as # of foci in each nucleus counted. Dot plots can be graphed and the mean, median, and significance of these data can be calculated.

## 4. Expected Results

**Figure 2:**
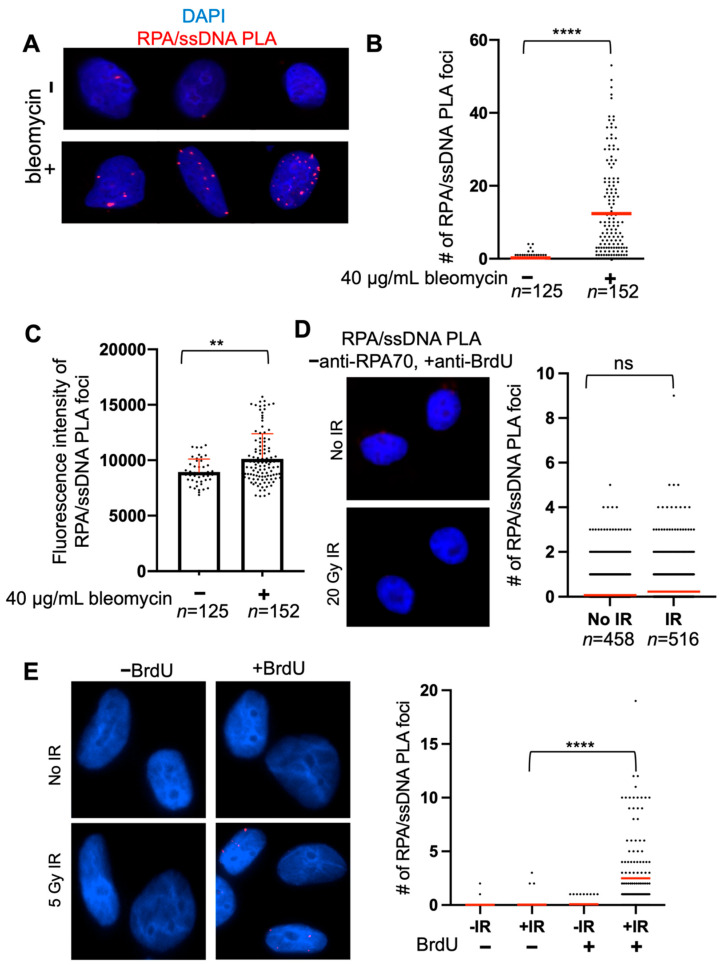
Quantification of RPA/ssDNA PLA foci. (**A**) Representative images of RPA/ssDNA PLA foci before and 2 h after bleomycin treatment. (**B**) Quantification of RPA/ssDNA PLA foci per nucleus before and 2 h after bleomycin treatment. For -bleomycin condition, red line indicates mean # of PLA foci= 0.23 and % of cells with >1 PLA foci = 15%. For +bleomycin condition, red line indicates mean # of PLA foci = 12.39 and % of cells with >1 PLA foci = 76% (**** *p* < 0.001, *t*-test). (**C**) Quantification of fluorescence intensity of RPA/ssDNA PLA foci from (**A**,**B**). Box represents mean fluorescence (−bleomycin = 8944, +bleomycin = 10,129), red error bar indicates standard deviation (** *p* < 0.01, *t*-test). (**D**) Representative images and quantification of RPA/ssDNA PLA foci negative control without anti-RPA70 antibody treatment before and 2 h after 20 Gy IR. Red line indicates mean # of RPA/ssDNA PLA foci (No IR = 0.1, IR = 0.2). (**E**) Representative images and quantification of RPA/ssDNA PLA foci control experiment in cells with and without BrdU incorporation, before and 2 h after 5 Gy IR. Red line indicates mean # of RPA/ssDNA PLA foci (−BrdU − IR = 0.02, *n* = 155; −BrdU + IR = 0.05, *n* = 152; +BrdU − IR = 0.07, *n* = 127; +BrdU + IR = 2.5, *n* = 153) (**** *p* < 0.001).

RPA/ssDNA PLA foci after bleomycin treatment should appear as discrete, bright foci (Figure 2A). PLA signal can coalesce, and, therefore, optimization is critical to observe discrete foci. In this example, the RPA/ssDNA foci number increases about 50-fold after bleomycin treatment, and it would be expected that the magnitude of this increase depends on timing and bleomycin dose (Figure 2B). Another way to quantify PLA foci data is to measure the % of cells with foci, i.e., % of cells with >0 RPA/ssDNA foci—in this way, we observe a five-fold increase in the % of cells with foci (Figure 2B).

A useful quantitative measure is the fluorescence intensity of the RPA/ssDNA PLA foci, because longer stretches of ssDNA will have more RPA/ssDNA PLA foci that may overlap and not appear as individual foci, depending on the resolution of the imaging. For example, if a RAD51 knockout was expected to increase RPA at DSBs, there are not more resected DSBs in the cell that would lead to an increase in the number of RPA/ssDNA foci, but this may lead to more RPA/ssDNA foci at each resected DSB which may overlap. In this way, counting discrete foci may not be representative of the biology. Using this approach, we measured the fluorescence intensity of RPA/ssDNA PLA foci and found an increase in PLA foci fluorescence intensity after bleomycin treatment compared to no treatment, indicating that bleomycin indeed creates more DSBs and, therefore, sites that will be bound by RPA, which leads to an RPA/ssDNA PLA foci signal that overlaps (Figure 2C). Including control experiments such as the one presented in Figure 2D,E can verify that the RPA/PLA assay is working specifically—here, we show an example of the RPA/PLA assay performed without including the anti-RPA70 antibody, showing that an extremely small amount of background PLA signal may occur but does not differ between experimental conditions (Figure 2D). We also show a control experiment comparing cells treated with and without BrdU, showing that the anti-RPA70/anti-BrdU PLA foci formation does not occur non-specifically (Figure 2E).

**Figure 3:**
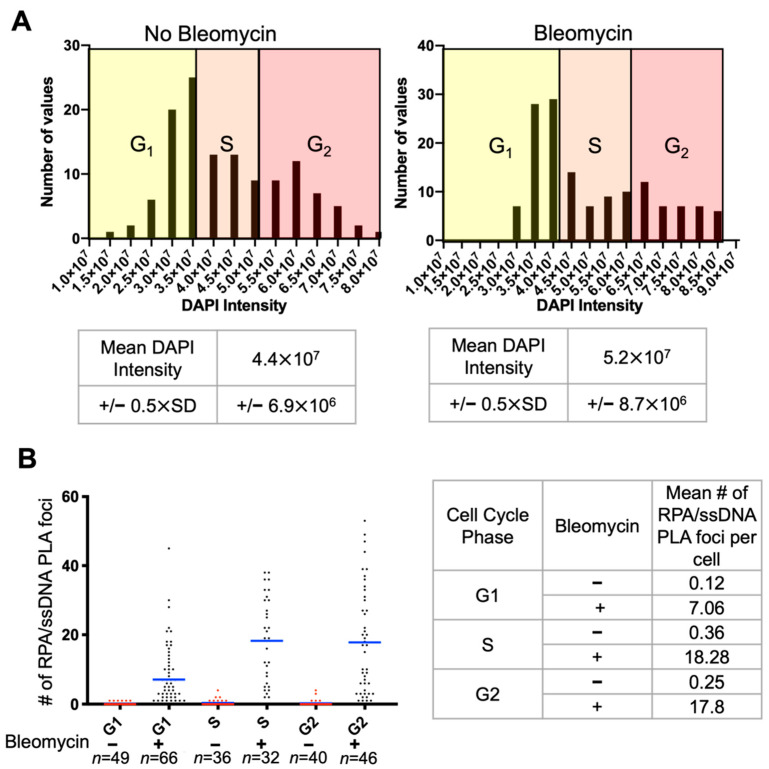
Cell cycle parsing of RPA/ssDNA PLA foci. (**A**) Example of parsing the cell cycle based on DAPI intensity. (**B**) Number of RPA/ssDNA PLA foci/ nucleus in each cell cycle phase before and 2 h after treatment with 40 μg/mL bleomycin.

Oftentimes, analysis of DNA end resection and homologous recombination is complicated by the fact that cells in the S phase actively undergoing replication have ssDNA bound by RPA. In order to analyze RPA/ssDNA at DSBs alone, the cell cycle can be parsed using DAPI intensity. Using this method is more accurate as more cells are analyzed, and each sample must be parsed on its own. The mean DAPI intensity + 0.5 × (Standard Deviation) provides an upper border for S phase cells, therefore DAPI intensity above this threshold indicates cells in G2. Mean DAPI intensity 0.5 × (Standard Deviation) is the lower threshold, and intensities below this are cells in G1 (Figure 3A). Though this method is not perfect, oftentimes there are not enough fluorescent channels to include a cell cycle marker, therefore this method provides a decent approximation. Using this method, we separated our RPA/ssDNA PLA foci into G1, S, and G2 phases. We see that cells in S and G2 have more foci than cells in G1, consistent with HR being preferentially used in these phases [22] (Figure 3B).

**Figure 4:**
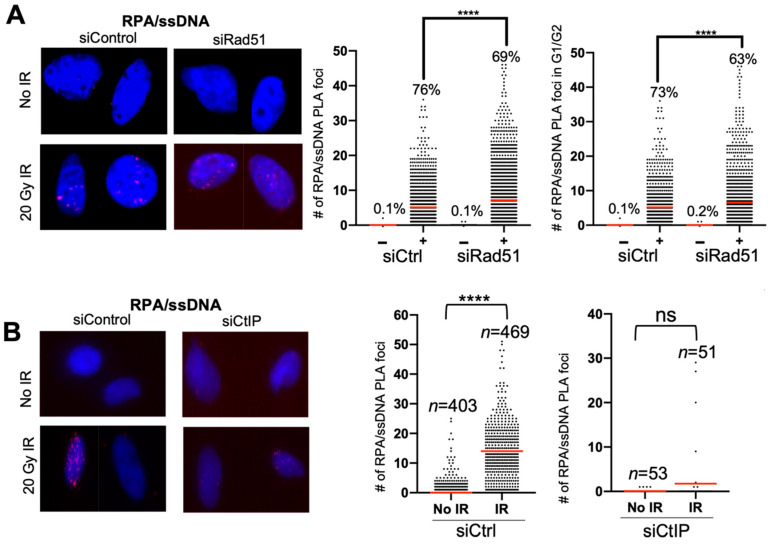
RPA/ssDNA foci analysis in RAD51- and CtIP-depleted cells after irradiation. (**A**) Representative images and quantification of RPA/ssDNA PLA foci before and 2 h after 20 Gy IR, with and without siRNA depletion of RAD51. Red line indicates average number of RPA/ssDNA PLA foci per nucleus (**** *p* value < 0.0001) and % indicates % of cells with at least 1 foci. siCtrl − IR *n* = 2377; siCtrl + IR *n* = 1256; siRad51 − IR *n* = 1628; siRad51 + IR *n* = 1583. (**B**) Representative images and quantification of RPA/ssDNA PLA foci before and 2 h after 20 Gy IR, with and without siRNA depletion of CtIP. Red line indicates average number of RPA/ssDNA PLA foci per nucleus (**** *p* value < 0.0001) Sample size of CtIP depleted cells is reduced due to some siRNA toxicity and fewer cells viable for PLA assay.

As proof-of-concept, we performed the RPA/ssDNA PLA experiment using irradiation as opposed to bleomycin, and with depletion of RAD51 and CtIP. Depletion of RAD51 leads to an increase in RPA on ssDNA, and CtIP depletion, which initiates DNA end resection and leads to a decrease in RPA foci [23,24,25]. We see that, after irradiation, the RPA/ssDNA PLA foci number increases dramatically, and, upon RAD51 depletion, there is an increase in the number of PLA foci (Figure 4A). Similarly, upon CtIP depletion, the number of RPA/ssDNA PLA foci is attenuated after IR (Figure 4B).

## 5. Conclusions

Here, we outline a PLA method to detect proteins associated with ssDNA during the DNA damage response. As an example, we show that RPA/ssDNA PLA foci are detectable and quantitative, and a wealth of information such as fluorescence intensity of foci and analysis of foci in specific cell cycle stages can be gathered. Using this method, more specific interactions than foci co-localization can be assessed and a fluorescent channel can be saved for additional analysis if desired.

One limitation to this method is the fact that PLA foci will form if the secondary oligos are within 40 nm. Because of this, detection of proteins on dsDNA at the dsDNA/ssDNA border may form a PLA interaction with ssDNA: for example, a PLA assay using anti-BrdU and anti-γH2A.X antibodies may form a PLA signal even though -γH2A.X is on dsDNA adjacent to ssDNA. Additionally, parsing the cell cycle in this way is imperfect. For example, cells assigned to G_1_ may actually be in the early S phase, etc. Nonetheless, it is a reasonable approximation that may be useful in studying HR repair and that excludes any possible interactions during replication.

Here, we tested this assay in U2OS cells, although other cell types may be possible to use in this assay. In U2OS cells, BrdU incorporation is not toxic, however, toxicity should be assessed in other cell types along with optimal BrdU concentrations.

## Data Availability

Not applicable.

## References

[1] Jackson S.P., Bartek J. (2009). The DNA-damage response in human biology and disease. Nature.

[2] Maser R.S., Monsen K.J., Nelms B.E., Petrini J.H. (1997). hMre11 and hRad50 nuclear foci are induced during the normal cellular response to DNA double-strand breaks. Mol. Cell. Biol..

[3] de Jager M., van Noort J., van Gent D.C., Dekker C., Kanaar R., Wyman C. (2001). Human Rad50/Mre11 is a flexible complex that can tether DNA ends. Mol. Cell.

[4] Syed A., Tainer J.A. (2018). The MRE11-RAD50-NBS1 Complex Conducts the Orchestration of Damage Signaling and Outcomes to Stress in DNA Replication and Repair. Annu. Rev. Biochem..

[5] Paull T.T. (2018). 20 Years of Mre11 Biology: No End in Sight. Mol. Cell.

[6] Mimitou E.P., Symington L.S. (2008). Sae2, Exo1 and Sgs1 collaborate in DNA double-strand break processing. Nature.

[7] Zhu Z., Chung W.H., Shim E.Y., Lee S.E., Ira G. (2008). Sgs1 helicase and two nucleases Dna2 and Exo1 resect DNA double-strand break ends. Cell.

[8] Gravel S., Chapman J.R., Magill C., Jackson S.P. (2008). DNA helicases Sgs1 and BLM promote DNA double-strand break resection. Genes Dev..

[9] Chen H., Lisby M., Symington L.S. (2013). RPA coordinates DNA end resection and prevents formation of DNA hairpins. Mol. Cell.

[10] San Filippo J., Sung P., Klein H. (2008). Mechanism of eukaryotic homologous recombination. Annu. Rev. Biochem..

[11] Sugiyama T., Kowalczykowski S.C. (2002). Rad52 protein associates with replication protein A (RPA)-single-stranded DNA to accelerate Rad51-mediated displacement of RPA and presynaptic complex formation. J. Biol. Chem..

[12] Beucher A., Birraux J., Tchouandong L., Barton O., Shibata A., Conrad S., Goodarzi A.A., Krempler A., Jeggo P.A., Löbrich M. (2009). ATM and Artemis promote homologous recombination of radiation-induced DNA double-strand breaks in G2. EMBO J..

[13] Zhou Y., Caron P., Legube G., Paull T.T. (2014). Quantitation of DNA double-strand break resection intermediates in human cells. Nucleic Acids Res..

[14] Canela A., Sridharan S., Sciascia N., Tubbs A., Meltzer P., Sleckman B.P., Nussenzweig A. (2016). DNA Breaks and End Resection Measured Genome-wide by End Sequencing. Mol. Cell.

[15] Forment J.V., Walker R.V., Jackson S.P. (2012). A high-throughput, flow cytometry-based method to quantify DNA-end resection in mammalian cells. Cytom. A.

[16] Nishi R., Wijnhoven P., le Sage C., Tjeertes J., Galanty Y., Forment J.V., Clague M.J., Urbé S., Jackson S.P. (2014). Systematic characterization of deubiquitylating enzymes for roles in maintaining genome integrity. Nat. Cell Biol..

[17] Mukherjee B., Tomimatsu N., Burma S. (2015). Immunofluorescence-based methods to monitor DNA end resection. Methods Mol. Biol..

[18] Jones T.R., Kang I.H., Wheeler D.B., Lindquist R.A., Papallo A., Sabatini D.M., Golland P., Carpenter A.E. (2008). CellProfiler Analyst: Data exploration and analysis software for complex image-based screens. BMC Bioinform..

[19] Held P. (2018). Monitoring Cell Cycle Progression in Cancer Cells.

[20] Roukos V., Pegoraro G., Voss T.C., Misteli T. (2015). Cell cycle staging of individual cells by fluorescence microscopy. Nat. Protoc..

[21] Ferro A., Mestre T., Carneiro P., Sahumbaiev I., Seruca R., Sanches J.M. (2017). Blue intensity matters for cell cycle profiling in fluorescence DAPI-stained images. Lab. Investig..

[22] Zhao X., Wei C., Li J., Xing P., Zheng S., Chen X. (2017). Cell cycle-dependent control of homologous recombination. Acta Biochim. Biophys. Sin..

[23] Ma C.J., Gibb B., Kwon Y., Sung P., Greene E.C. (2017). Protein dynamics of human RPA and RAD51 on ssDNA during assembly and disassembly of the RAD51 filament. Nucleic Acids Res..

[24] You Z., Shi L.Z., Zhu Q., Wu P., Zhang Y.W., Basilio A., Tonnu N., Verma I.M., Berns M.W., Hunter T. (2009). CtIP links DNA double-strand break sensing to resection. Mol. Cell.

[25] Sartori A.A., Lukas C., Coates J., Mistrik M., Fu S., Bartek J., Baer R., Lukas J., Jackson S.P. (2007). Human CtIP promotes DNA end resection. Nature.

